# Proteome metabolome and transcriptome data for three Symbiodiniaceae under ambient and heat stress conditions

**DOI:** 10.1038/s41597-022-01258-w

**Published:** 2022-04-05

**Authors:** Emma F. Camp, Tim Kahlke, Brandon Signal, Clinton A. Oakley, Adrian Lutz, Simon K. Davy, David J. Suggett, William P. Leggat

**Affiliations:** 1 Climate Change Cluster, University of Technology Sydney, Sydney, Australia; 2 School of Medicine, College of Health and Medicine, University of Tasmania, Hobart, Australia; 3 School of Biological Sciences, Victoria University of Wellington, Wellington, New Zealand; 4 Metabolomics Australia, Bio21 Institute, The University of Melbourne, Parkville, Australia; 5 School of Environmental and Life Sciences, University of Newcastle, Callaghan, Australia

**Keywords:** Marine biology, Cellular microbiology

## Abstract

The Symbiodiniaceae are a taxonomically and functionally diverse family of marine dinoflagellates. Their symbiotic relationship with invertebrates such as scleractinian corals has made them the focus of decades of research to resolve the underlying biology regulating their sensitivity to stressors, particularly thermal stress. Research to-date suggests that Symbiodiniaceae stress sensitivity is governed by a complex interplay between phylogenetic dependent and independent traits (diversity of characteristics of a species). Consequently, there is a need for datasets that simultaneously broadly resolve molecular and physiological processes under stressed and non-stressed conditions. Therefore, we provide a dataset simultaneously generating transcriptome, metabolome, and proteome data for three ecologically important Symbiodiniaceae isolates under nutrient replete growth conditions and two temperature treatments (ca. 26 °C and 32 °C). Elevated sea surface temperature is primarily responsible for coral bleaching events that occur when the coral-Symbiodiniaceae relationship has been disrupted. Symbiodiniaceae can strongly influence their host’s response to thermal stress and consequently it is necessary to resolve drivers of Symbiodiniaceae heat stress tolerance. We anticipate these datasets to expand our understanding on the key genotypic and functional properties that influence the sensitivities of Symbiodiniaceae to thermal stress.

## Abstract (summary)

**Table:** 

Measurement(s)	RNA sequences and counts • Metabolite abundance • Protein abundance
Technology Type(s)	Illumina HiSeq. 2500 • QTOF MS system • Orbitrap Fusion Lumos
Sample Characteristic - Organism	Symbiodiniaceae
Sample Characteristic - Environment	In culture

## Background & Summary

The Symbiodiniaceae are a family of marine dinoflagellates that are genetically and functionally diverse. Recent advancements in molecular analysis have revealed immense, previously undetectable genetic diversity^1–4^. The family now has ten recognised genera, and five equivalent lineages yet to be formally described^1,3–6^. Symbiodiniaceae are found across a broad range of environments that span temperate and tropical biomes^3^. They also have various life-histories that include free-living^7–9^, diverse substrate attachment^8^, and a symbiotic state with invertebrates, e.g., scleractinian coral^3^. The symbiosis between Symbiodiniaceae and coral forms the foundation of coral reefs, aiding their survival and development in tropical, nutrient-poor waters^10^. In turn, dysbiosis of Symbiodiniaceae and corals under stressful environmental conditions, e.g., warming seawater from climate change, can compromise the survival of reef ecosystems. Loss of Symbiodiniaceae from the coral host compromises the host’s access to essential nutrients^11–13^ and metabolites^14^, and results in visual paling, commonly referred to as coral bleaching^15^. More frequent and intense marine heatwaves in combination with increasing mean sea surface temperatures are resulting in more frequent and severe global coral bleaching events that can result in mass coral mortality^16,17^. The species (or genotype) of Symbiodiniaceae hosted can significantly influence the coral response to thermal stress^18^, and so specificity of Symbiodiniaceae-coral host associations often reflects ecological patterns of thermally induced coral bleaching^19,20^. Consequently, the focus of many global research efforts has been on trying to understand the molecular, ecological, and biogeochemical factors influencing sensitivity of these symbioses.

Isolation and *in vitro* culturing of Symbiodiniaceae has become a widely adopted tool to further understanding of coral sensitivity to stressors^18^. In some instances, phylogenetic differences between isolates of Symbiodiniaceae have been documented to explain their stress response^21^. However, broader-scale studies inter-comparing multiple Symbiodiniaceae taxa are increasingly demonstrating the complex interplay between phylogenetic-dependent and -independent traits (diversity of characteristics of a species^22^) that govern Symbiodiniaceae stress responses. Functional “types” have thus been recognised as a key operational unit determining the stress response, and ultimate ecological success of Symbiodiniaceae^23–25^. Even so, the challenge remains identifying the most appropriate suite of both molecular and physiological traits to best define Symbiodiniaceae functional types. Furthermore, to truly understand the organismal response to stress, knowledge of the entire biological system is required, beyond just discrete trait properties^26^. This is where metabolic network analysis that applies multiple omics methods is beneficial, as it can begin to uncover any cross talk in traits and networks that ultimately govern the Symbiodiniaceae stress response.

To date, measuring photosynthetic functioning, particularly photosystem II (PSII) maximum photochemical efficiency (*F*_*v*_*/F*_*m*_), has been broadly applied to assess stress sensitivity of cultured Symbiodiniaceae^18^. Technological advancements and improved cost-effectiveness of omics analysis have led to more studies integrating omics techniques into their experimental designs to assess the stress response of cultured Symbiodiniaceae^27^. Studies have considered the transcriptional response of cultured Symbiodiniaceae to varying light regimes^28^, pH conditions^29^ and heat stress^21,29–33^. For example, Levin *et al*.^21^ ran a 13-day heat stress (32 °C) experiment on a thermo-tolerant *Cladocopium* sp. The authors observed no sign of physiological stress (e.g., stable *F*_*v*_*/F*_*m*_), but transcriptomics analysis revealed upregulation (by ≥ 4-fold) of reactive oxygen species (ROS) scavenging, and molecular chaperone genes^21^. Evidence that Symbiodiniaceae employ significant post-transcriptional and post-translational modification for gene regulation^34,35^ makes other omics techniques, such as proteomics and metabolomics essential. Recent proteomics analysis on heat stressed *Breviolum psygmophilum* found several hypothesised relevant surface proteins that were differentially expressed^36^. Integrating omics methods, e.g., transcriptomics and metabolomics^14^, is significantly advancing our understanding of both the molecular and physiological traits that ultimately govern the Symbiodiniaceae stress response.

In this study, we determined simultaneous changes in the transcriptome, metabolome, and proteome of three Symbiodiniaceae isolates under nutrient replete growth conditions and two temperature treatments (ca. 26 °C and 32 °C). Concurrent measurements of Symbiodiniaceae-specific bacteria were also made and have previously been published in Camp *et al*.^37^. While previous studies have considered the transcriptional e.g.^21,29–33^ and metabolite e.g.^14,38,39^ profiles of Symbiodiniaceae, the proteome of Symbiodiniaceae is still in its infancy despite its profound ability to resolve functional diversity in other microalgae^36^. Transcriptomics has furthered knowledge at a cellular level e.g.^21,29–33^, but it is metabolomics and proteomics techniques that provide knowledge on the outcomes of cellular processes^36,38^. No known datasets exist that contain uniformly generated and processed transcriptome, metabolome, and proteome datasets, required for a systems biology approach to resolving different functional responses occurring in Symbiodiniaceae across different temperature profiles. The presented datasets will provide a fundamental understanding on the genotypic and functional variance of Symbiodiniaceae isolates to thermal stress.

## Methods

### Symbiodiniaceae culture conditions

**Fig. 1:**
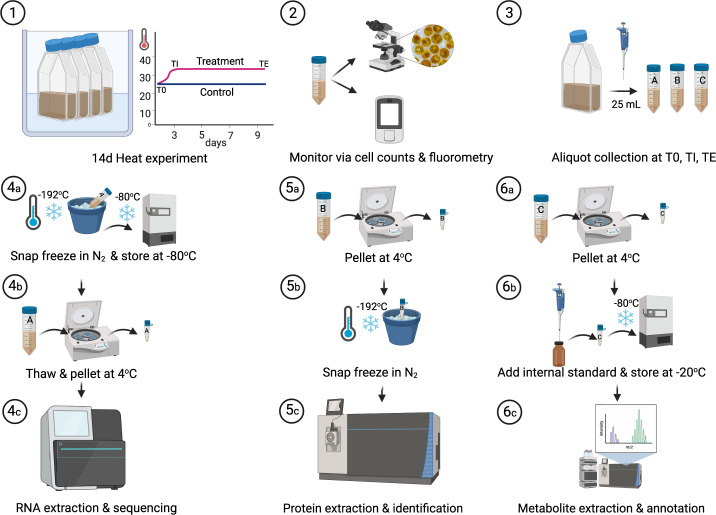
Experimental scheme. Three Symbiodiniaceae isolates (C1, *Cladocopium goreaui*, (identifier: C1-124), D1a, *Durusdinium trenchii*, B1, *Breviolum* sp.) were grown in replicate (*n* = 4) at 26 °C and 32 °C (1). Culture health was regularly monitored via cell counts and fluorometry (see Table 2) (2). At three time points, 25 mL × 3, per culture was removed for subsequent analysis (3). For RNA extraction and sequencing, the culture aliquot was immediately snap-frozen and stored at −80 °C (4a), prior to thawing and pelleting (4b), and RNA extraction (4c). For protein extraction, culture aliquots were pelleted at 4 °C (5a), before snap-freezing in liquid nitrogen (5b) ahead of subsequent extraction protocols (5c). For metabolite extraction, culture aliquots were pelleted at 4 °C (6a). Pellets were then re-suspended with an internal standard (analytical grade 0.005 mM 2-Aminoanthracene) and stored at −80 °C (6b) prior to metabolite extraction protocols (see methods text) (6c).

Three Symbiodiniaceae cultures, ITS2 isolate C1 (*Cladocopium goreaui*, culture identifier: C1-124), ITS2 isolate D1a (*Durusdinium trenchii*), and ITS2 isolate B1 (*Breviolum* sp.) were obtained from the long-term laboratory stock at the University of Technology Sydney (UTS) (see Table 1 for further information) grown under 20.0 °C. All isolates had been maintained in culture for over a decade. Isolates were moved to 26.0 °C and were grown for three months prior to the experimentation. Isolates were cultured in Daigo IMK medium (Nihon Pharmaceutical Co. Ltd.), kept in 750 mL culture flasks grown under a white light of 207 ± 0.05 µmol photons m^−2^ s^−1^ on a 12:12 h light:dark cycle. Light was provided by three Hydra 52 HD LED units (Aqua Illumination, Ames). Cultures were grown for ten days under two temperature conditions (mean ± SE), 26.0 ± 0.5 °C (Control) and 32.4 ± 0.01 °C (Treatment). Temperature was ramped 2 °C per day for three consecutive days to achieve 32.4 °C. Temperatures were achieved by water baths controlled with Julabo heaters (JULABO GmbH). All cultures were maintained in exponential growth for subsequent sample analysis, and culture cell densities were maintained via regular dilutions at ca. 150,000 cells mL^−1^ to prevent carbon or nutrient limitation^39^. Health of the isolates was monitored via cell counts and fluorometry (maximum quantum yield of PSII (*F*_*v*_*/F*_*m*_)), with both cell counts and *F*_*v*_*/F*_*m*_ declining in the heat treatment^37^ (Table 2). Samples were collected at three points in time, prior to temperature ramping (day 0, T0), after temperature ramping (day 3, TI), and then at the end of seven days (day 10, TE, Fig. 1). All cultures had four biological replicates across each temperature condition (Table 3), however not all samples were able to be processed for their respective analyses due to cost, time, and availability of biological material; final numbers are presented in Table 4. During sample processing, culture flasks were systematically removed from the water baths to minimise time out of the experimental conditions. One replicate flask of each isolate for control and treatment was taken at a time, creating four sampling blocks of six culture flasks, each sampled an hour apart. For each of the four sampling blocks, the order in which a culture was sampled first was rotated (see Table 3 and Camp *et al*.^37^).

**Table 1:** Isolate information.

Genus	Species	ITS2 Major type profile	Culture isolate identity	Internal isolate label	Geographic origin	Host isolate
*Breviolum*	sp.	B1	B (UTS)	B1-UTS-B	South Taiwan (Indo-Pacific)	*Euphyllia glabrescens* (coral)
*Cladocopium*	*goreaui*	C1	SCF055-06	C1-124/SCF124	Magnetic Island (Pacific)	*Acropora tenuis* (coral)
*Durusdinium*	* trenchii*	D1a	amur‐D‐MI, UTS-D, (UTS_D)	SCF082	Magnetic Island (Pacific)	*Acropora muricata* (coral)

Information on the Symbiodiniaceae origin (host isolate or free-living and geographic location) and ITS2 major type profile. Culture isolate identification is also provided as found in the literature and as labelled internally at the University of Technology of Sydney (UTS).

**Table 2:** Cell counts and maximum quantum yield of PSII (*F*_*v*_*/F*_*m*_).

Isolate	Time Point	Treatment	Cell mL^−1^		*F*_*v*_*/F*_*m*_	
			Mean	SE	Mean	SE
D1a	T0	Control	164134	8436	0.55	0.01
	TE		126282	12680	0.43	0.01
	T0	Treatment	173596	17762	0.53	0.00
	TE		78151	7263	0.36	0.00
B1	T0	Control	156750	8413	0.53	0.00
	TE		155405	19992	0.51	0.01
	T0	Treatment	131500	19506	0.53	0.01
	TE		72441	4191	0.10	0.03
C1-124	T0	Control	138675	8405	0.59	0.02
	TE		121877	18468	0.50	0.00
	T0	Treatment	122000	19877	0.58	0.02
	TE		49925	3544	0.33	0.02

Data is expressed as means ± SE (n = 4) for each Symbiodiniaceae isolate (*Breviolum* sp. (B1), *Cladocopium goreaui* (C1-124), and *Durusdinium trenchii* (D1a)) at day 0, (T0), and at the end of the experiment (day 10, TE).

**Table 3:** Sample identification and processing order.

Sub-sample order	Sample ID	Experimental condition	Isolate
Round-1	1	Control	D1a
	17	Treatment	D1a
	9	Control	B1
	25	Treatment	B1
	13	Control	C1-124
	29	Treatment	C1-124
Round-2	10	Control	B1
	26	Treatment	B1
	14	Control	C1-124
	30	Treatment	C1-124
	2	Control	D1a
	18	Treatment	D1a
Round-3	11	Control	B1
	27	Treatment	B1
	15	Control	C1-124
	31	Treatment	C1-124
	3	Control	D1a
	19	Treatment	D1a
Round-4	16	Control	C1-124
	32	Treatment	C1-124
	4	Control	D1a
	20	Treatment	D1a
	12	Control	B1
	28	Treatment	B1

A systematic design was used to process samples in four rounds to ensure one replicate of each Symbiodiniaceae isolate and experimental condition. This design was undertaken to account for processing time. The sample ID is used throughout as an identifier, along with the experimental condition (control maintained at ca. 26 °C and treatment maintained at ca. 32 °C) and time points (day 0, (T0), after temperature ramping (day 3, TI), and then at the end of seven days (day 10, TE) (see methods and Table 3).

**Table 4:** Information on the data reposited for each method within the experimental set-up.

Isolate	Time-point	Treatment or Control	Transcriptomics	Metabolomics	Proteomics
*Cladocopium goreaui*	T0	Control	n = 4*	n = 4	n = 3
*Durusdinium trenchii*			n = 4*	n = 4	n = 2
*Breviolum* sp.			n = 4*	n = 4	n = 3
*Cladocopium goreaui*		Treatment	n = 4*	n = 4	n = 1
*Durusdinium trenchii*			n = 4*	n = 4	n = 1
*Breviolum* sp.			n = 4*	n = 4	n = 1
*Cladocopium goreaui*	TI	Control	n = 4	n = 4	n = 1
*Durusdinium trenchii*			n = 4	n = 4	n = 2
*Breviolum* sp.			n = 4	n = 4	n = 1
*Cladocopium goreaui*		Treatment	n = 4	n = 4	n = 4
*Durusdinium trenchii*			n = 4	n = 4	n = 4
*Breviolum* sp.			n = 4	n = 4	n = 4
*Cladocopium goreaui*	TE	Control	n = 4	n = 4	n = 1
*Durusdinium trenchii*			n = 4	n = 4	n = 1
*Breviolum* sp.			n = 4	n = 4	n = 0
*Cladocopium goreaui*		Treatment	n = 4	n = 4	n = 4
*Durusdinium trenchii*			n = 4	n = 4	n = 4
*Breviolum* sp.			n = 4	n = 4	n = 4

*Indicates that the raw sequence files were uploaded but they are not included in the paper analyses. Samples were collected at three points in time, prior to temperature ramping (day 0, T0), after temperature ramping (day 3, TI), and then at the end of seven days (day 10, TE).

### RNA extraction and sequencing

For each replicate, a 25 mL aliquot of culture was removed in a sterile culturing facility at UTS and immediately snap frozen in liquid nitrogen. Preliminary testing by microscopy analysis confirmed no visual signs of cell lysis. After collection, the frozen cells were thawed at room temperature in a water bath and pelleted through centrifugation for 5 min at 3,000 *g* and at 4°C. The supernatant was discarded, and the remaining pellet re-suspended using 450 µL of RLT buffer solution (RNeasy Plant Mini Kit, Qiagen, Hilden, Germany) and 4.5 µL of β-mercaptoethanol (Sigma-Aldrich, Australia). The pelleted cells were lysed by bead beating with 0.2 *g* of 0.5 mm sterile acid-washed glass beads (Biospec, OK, USA) in the TissueLyser II (Qiagen, Australia) at 50 Hz for 2 min. The lysate was then used for RNA extraction and purification using the Rneasy mini plant kit (Qiagen, Australia). A total volume of 5 µL of extracted sample RNA was eluted into Nuclease-free water (Sigma-Aldrich, Australia) and subsequently stirred at −80 °C before sequencing.

RNA quality and yield (150–500 ng) were checked using a Bioanalyser Agilent 2100 (Agilent Technologies, CA, USA). RNA was polyA-enriched and sequencing libraries were prepared using Illumina’s TruSeq stranded library preparation kit and sequenced on an HiSeq2500 Sequencer (Illumina, CA, USA) resulting in a total of 793,531,730 paired-end sequencing reads (Supplementary Tables 1–3). Sequencing was performed at the Australian Genome Research Facility, Melbourne, Australia.

### RNASeq data processing and bioinformatic analysis

All samples were processed as described in^21^. In summary, raw fastq files of all samples (NCBI SRA bioproject PRJNA723630) were processed with Trim Galore! (v0.6.0; Babraham Bioinformatics) with default settings to remove low quality sequences as well as sequencing adapters. Trimmed fastq files were combined into a single fastq for each Symbiodiniaceae isolate and assembled into transcripts using Trinity (v2.8.4) in *de novo* mode with default parameters^40^. Gene expression quantification for each sample was performed using kallisto v0.43.1^41^ in conjunction with the Trinity script *align_and_estimate_abundance.pl*. Counts for each gene were calculated by combining the read counts for each gene isoform. Open Reading Frames (ORFs) were predicted in the assembled transcripts using Transdecoder v5.5.0 (https://github.com/Transdecoder)^40^ with default settings. Predicted protein sequences were filtered by length and proteins <100 amino acids were removed prior to functional annotation. The remaining protein sequences were annotated using InterproScan v5.27^42^. Differential expression analysis was performed in R using voom^43^ and limma^44^ on gene read counts. Prior to normalisation, genes were filtered by read count. We analysed control and treatment samples of each time point together. For each time point, genes with less than ten reads in at least four samples were removed from the analysis. Reads were normalised using voom, with normalisation factors being calculated with calcNormFactors(). For each comparison (Treatment TE vs Control TE, and Treatment TI vs Control TI), linear models were fitted using lmFit(), contrasts for each gene estimated using contrasts.fit(), and empirical Bayes smoothing of standard errors performed using eBayes().

### Protein extraction, identification, and quantification

For each replicate, a 25 mL aliquot of culture was removed in a sterile culturing facility at UTS. Cells were pelleted through centrifugation for 5 min at 3,000 *g* and at 4 °C. The medium was discarded, and the pellet stored at −80 °C. For each species, the control consisted of all samples from the initial timepoint (T0) as well as the 26 °C samples from later timepoints (TI and TE). Proteins were extracted, digested, and purified using a modified filter-aided sample preparation protocol^45^, as follows. Salt was removed by centrifugation and resuspension of the cells in liquid chromatography-grade water twice, followed by transfer to low protein binding tubes (Eppendorf LoBind, Eppendorf SE) and freezing at −80 °C until further processing. The algal pellet was then resuspended in 500 μL 5% w/v sodium deoxycholate and the cells lysed by 20 × 2 s pulses with an ultrasonicator probe. Proteins were reduced with 1% final concentration β-mercaptoethanol and denatured by incubation at 85 °C for 30 min. Photosynthetic pigments were partially removed by twice adding two volumes of ethyl acetate, vortexing for 1 min, and centrifugation at 10,000 *g* × 1 min, discarding the upper organic fraction each time. Residual ethyl acetate was removed by centrifugation under vacuum for 15 min. The supernatant was then transferred to a centrifugation filter (Amicon Ultra 30 kDa 0.5 mL, Merck Millepore) and concentrated by centrifugation (14,000 *g* × 20 min). Proteins were then resuspended and washed twice by the addition of 380 μL 50 mM Tris buffer, pH 8.1, followed by centrifugation (14,000 *g* × 15 min). After a third and final addition of 380 μL 50 mM Tris buffer, a 10 μL subsample was transferred to a new tube, diluted with 90 μL water (Optima LC/MS Grade, ThermoFisher), acidified with 1 μL 100% formic acid to precipitate deoxycholate, centrifuged (16,000 *g* × 5 min) and the protein quantified by protein-binding dye fluorescence (Qubit 2.0, ThermoFisher Scientific, USA). A total of 100 μg total protein were reduced by incubation with 10 mM β-mercaptoethanol at 37 °C for 10 min, alkylated by incubation for 20 min at room temperature with 20 mM acrylamide, the alkylation quenched by a second equal addition of β-mercaptoethanol, and digested with 2 μg trypsin for 18 h in the filter unit. Digested peptides were collected by centrifugation (14,000 *g* × 20 min) through the filter and any remaining deoxycholate removed by the addition of formic acid to 1% final concentration, followed by centrifugation (16,000 *g* × 1 min). The supernatant, containing digested peptides, was transferred to a new tube, concentrated by vacuum centrifugation to approximately 200 μL and desalted using C18-packed pipette tips (Omix Bond Elut, Agilent Technologies). Peptides were dried by centrifugation under vacuum and stored at 4 °C until analysis, upon which they were dissolved in 70 μL 0.1% formic acid and the peptide content quantified by fluorescence as before. Unless otherwise noted, all reagents were obtained from Sigma-Aldrich New Zealand, and all samples were handled in low protein binding tubes (Eppendorf).

Peptide samples were separated by a 75 min linear gradient from 5%/95% to 35%/65% buffer A/B (buffer A: 0.1% formic acid; buffer B: 80% acetonitrile, 0.1% formic acid) at 300 nL min^−1^ on an Acclaim PepMap C18, 3 μm, 100 Å column (Thermo Scientific, Auckland, New Zealand) and Ultimate 3000 liquid chromatograph system (Dionex, Sunnyvale, CA). Peptides were ionised by electrospray at 1.8 kV and analysed by an Orbitrap Fusion Lumos Tribrid mass spectrometer (Thermo Scientific). Precursor mass spectra were acquired in the Orbitrap at a resolution of 120,000, rejecting singly-charged ions, with quadrupole isolation enabled and an automatic gain target of 7.0e5 and a maximum injection time of 50 ms. The 20 most intense precursor spectra were fragmented by higher-energy collision dissociation and analysed in the ion trap with an automatic gain target of 5.0e3 and a maximum injection time of 50 ms. Dynamic exclusion was enabled with a duration of 60 s. Samples were each analysed 1–3 times (depending on available biomass) as technical replicates in a stratified random order.

Proteins were identified from raw spectrum files using the Andromeda search algorithm in MaxQuant 1.6.10.43^46,47^. Protein search databases were generated for each Symbiodiniaceae species from transcripts obtained from samples from the same experiment. Trypsin was the selected digest enzyme with a maximum of two missed cleavages allowed. Oxidation of methionine and acetylation of the protein n-terminus were considered as variable modifications and carbamidomethylation of cysteine considered as a fixed modification. The first search and main search peptide tolerances were 20 ppm and 4.5 ppm, respectively, and a mass tolerance of 0.5 Da for the ion trap MS2 search parameter. Label-free quantification was enabled with a minimum of two unique plus razor peptides for quantification and match between runs enabled. Peptide spectrum match and protein false discovery rates were both set to 1%, with a minimum of two peptides required for annotation. Three MaxQuant analyses, one for each species, were conducted separately using the same settings and the search database specific to each species.

### Metabolomic extraction, annotation, and quantification

For each replicate, a 25 mL aliquot of culture was removed in a sterile culturing facility at UTS. Cells were pelleted through centrifugation (5 min at 3,000 *g* and at 4 °C). To each sample, 150 µL of 100% cold MeOH (spiked with the internal standard 0.005 mM 2-aminoanthracene (technical grade, Sigma Aldrich, Australia) were added and the pellet re-suspended in a scintillation vial. Vials were covered in tin foil to prevent light degradation and placed for 24 h in −80 °C storage. Samples were then homogenised using a TissueLyser LT (Qiagen Inc., Hilden, Germany) with 20 mg acid washed glass beads (425–600 µm; Sigma Aldrich, Australia). Visual counts (via a haemocytometer) of trial samples confirmed effective cellular disruption of over 90% of cells^48^. Samples were pelleted (5 min at 3,000 *g* and at 4 °C) and the supernatant collected and placed on ice. The remaining pellet was resuspended with 100 µL of 100% MeOH (again spiked with the internal standard) and again pelleted (5 min at 3,000 *g* and at 4 °C). The subsequent supernatant was combined with the supernatant from the first round and stored in a tin foil covered scintillation vial at −80 °C for 7 days prior to analysis.

Untargeted LC-MS profiling analysis of samples was carried out by Metabolomics Australia (School of BioSciences, University of Melbourne). Instrument and LC-MS setup were as follows: Agilent 6520 QTOF MS system (Agilent Technologies, Santa Clara, CA, USA) with a dual sprayer ESI source and attached to Agilent 1200 series HPLC system comprised of a vacuum degasser, binary pump, thermostated auto-sampler and column oven. The MS was operated in positive mode using the following conditions: nebulizer pressure 30 psi, gas flow-rate 10 L min^−1^, gas temperature 300 °C, capillary voltage 4000 V, fragmentor 150 and skimmer 65 V. Instrument was operated in the extended dynamic range mode with data collected in m/z range 70–1700. Chromatography was carried out using an Agilent Zorbax Eclipse XDB-C18, 2.1 × 100 mm, 1.8 µm column maintained at 40 °C ( ± 1 °C) at a flow rate of 400 µL min^−1^ with a 20 min run time. A gradient LC method was used with mobile phases comprised of (A) 0.1% formic acid in deionized water and (B) 0.1% formic acid in acetonitrile: 5 min linear gradient from 5% to 30% mobile phase B, followed by a 5 min gradient to 100% mobile phase B and then a 5 min hold, followed by a 5 min re-equilibration at 5% mobile phase B. Molecular feature extraction (MFE) was conducted in R using Bioconductor^49^.

**Fig. 2:**
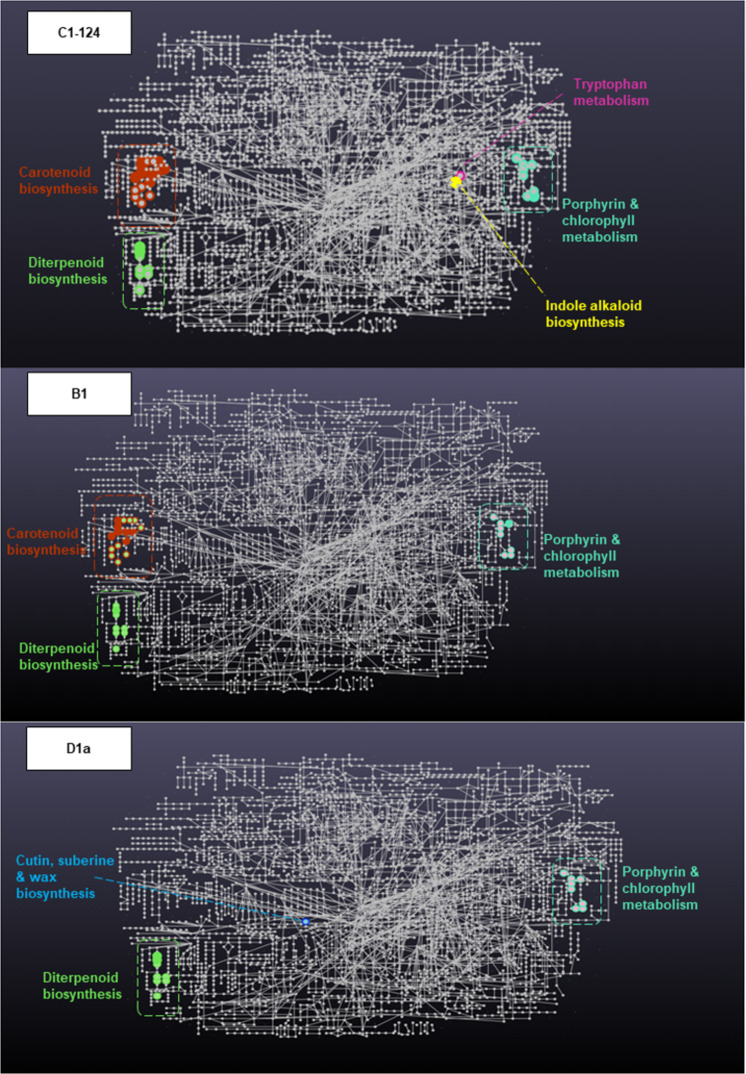
Metabolite pathway analysis at the end time-point (TE) for the three Symbiodiniaceae isolates (C1, *Cladocopium goreaui*, (identifier: C1-124), D1a, *Durusdinium trenchii*, B1, *Breviolum* sp.). Functional analysis using mummichog and a top-10% peak cut-off was undertaken in Metaboanalyst^51^. Data were median-normalised and log-transformed. For each isolate, TE control (*n* = 4 maintained at ca. 26 °C) versus treatment (*n* = 4 maintained at ca. 32 °C) were analysed. Results are visualised in a KEGG format pathway analysis that were searched against *Arabidopsis thaliana* (the best photosynthetic organism match based on available options and high pathway coverage). The coloured pathways indicated significant differences between the two treatments.

Confident annotation of metabolites from reverse phase untargeted LC-MS profiling is a significant challenge for non-model organisms^50^. We thus eschewed a direct annotation, and utilised the mummichog-style analysis in Metaboanalyst 5.0^51^ to provide a glimpse of pathways amenable to reverse phase metabolomic profiling. In contrast to direct annotation, such functional analysis leverages the organization of metabolic networks to predict functional activity directly from feature tables, bypassing metabolite identification. Thus, high-quality hypotheses can be quickly generated from an LC-MS peak table^52^. Functional analysis using mummichog and a top-10% peak cut-off was undertaken in Metaboanalyst^51^. Data were median-normalised and log-transformed. For each isolate, TE control (*n* = 4) *versus* treatment (*n* = 4) were analysed. Results were visualised in a KEGG format pathway analysis that were searched against *Arabidopsis thaliana* (the best photosynthetic organism match based on available options and high pathway coverage). The coloured pathways indicated significant differences between the two treatments (Fig. 2).

## Data Records

All data are reposited with an identification format that includes the sample ID (indicated in Table 3), and experimental time point of collection prior to temperature ramping (day 0, T0), after temperature ramping (day 3, TI), and then at the end of seven days (day 10, TE). Table 3 denotes whether a sample is a control (C) or treatment (T).

Mass spectrometry proteomics data have been deposited to the ProteomeXchange Consortium via the PRIDE partner repository^53^ with the dataset identifiers PXD025080 (*Breviolum* sp.)^54^, PXD025051 (*C. goreaui*)^55^ and PXD025050 (*D. trenchii*)^56^. For proteomic data, the PRIDE data repository also has dates indicated, e.g. “18_11–19” that represent the dates (Day-Month_Year) that the peptide sample were analysed by the mass spectrometer.

Raw RNASeq data has been deposited into the Sequence Read Archive (SRA) at the National Center for Biotechnology Information under bioproject PRJNA723630^57^.

Furthermore, differential gene expression results, normalised read counts, functional annotation of assembled RNA transcripts and raw metabolomics data sets of all time points have been deposited into the Open Science Forum project “*Metabolomics data and differential gene expression results or three coral symbionts (Cladocopium goreaui, Durusdinium trenchii, and Breviolum sp.) under elevated temperature stress*.^58^”.

## Technical Validation

Re-verification of the Symbiodiniaceae culture genotypes was conducted on the stock aliquots prior to sub-culturing. A 5 mL aliquot was removed and pelleted through centrifugation for 5 min at 3,000 g. The supernatant was discarded, and the pellet went through two wash steps with PBS and repeat centrifugation. The excess PBS was removed, and the remaining pellet resuspended in 410 µL of Bead Solution (Qiagen, Australia) and 40 µL of Phenolic separation solution (Qiagen, Australia). Cells were subsequently lysed by bead beating with 0.2 g of 0.5 mm sterile acid-washed glass beads (Biospec, OK, USA) in the TissueLyser II (Qiagen, Australia) at 50 Hz for 2 min. The lysate was then used for DNA extraction and purification using the PowerPlant kit (Qiagen, Australia). Polymerase chain reaction (PCR) amplification of the ITS2 region was performed using the primers ITSintfor2 and ITS2-reverse following the PCR conditions of Arif *et al*.^59^. Purified PCR products were sequenced by the Australian Genome Research Facility.

The transcript-based protein search databases were analysed with BUSCO (v5.2.2), see Table 5. The complete BUSCO values range from 77.3–80.8% (Table 5), which are comparable to other datasets in non-model organisms (70–92% in 49 taxa across multiple invertebrate animal phyla^60^). From this analysis and the high number of identified proteins, we conclude that these search databases are appropriate and of high quality.

**Table 5:** The transcript-based protein search databases results analysed with BUSCO (v5.2.2).

	*Breviolum* sp.		*Cladocopium goreaui*		*Durusdinium trenchii*	
Complete BUSCOs	200	78.4%	197	77.3%	206	80.8%
Complete and single-copy BUSCOs	160	62.7%	169	66.3%	152	59.6%
Complete and duplicated BUSCOs	40	15.7%	28	11.0%	54	21.2%
Fragmented BUSCOs	13	5.1%	14	5.5%	13	5.1%
Missing BUSCOs	42	16.5%	44	17.2%	36	14.1%
Total searched	255		255		255	

The complete BUSCO values range from 77.3–80.8%, which are comparable to other datasets in non-model organisms (70–92% in 49 taxa across multiple invertebrate animal phyla^59^). From this analysis and the high number of identified proteins we conclude that these search databases are appropriate and of high quality.

## Supplementary information

Supplementary Materials

## Data Availability

The version and parameter of all bioinformatics tools used in this work are described in the Methods section.
