# Supplementary material for: Proteome metabolome and transcriptome data for three Symbiodiniaceae under ambient and heat stress conditions
Source: Sci Data. 2022 Apr 5;9:153. doi: 10.1038/s41597-022-01258-w (PMC8983644; doi:10.1038/s41597-022-01258-w)
Supplement: Supplementary file 1 — Supplementary Materials [file 41597_2022_1258_MOESM1_ESM.docx]

**Supplementary Materials**

**Proteome metabolome and transcriptome data for three Symbiodiniaceae under ambient and heat stress conditions**

Emma F. Camp^1^*, Tim Kahlke^1^, Brandon Signal^2^, Clinton A. Oakley^3^, Adrian Lutz^4^, Simon K. Davy^3^, David J. Suggett^1^, William P. Leggat^5^

**Tables**

**Table S1. Summary Statistics RNASeq data for the Symbiodiniaceae isolate *Breviolum* sp. (B1)** Sample column demotes the sample ID, which indicates sampling timepoint (start (T0), first time point (TI) or end time point (TE))-control or treatment-replicate-read direction (forward for R1 and reverse for R2). Control was maintained at ca. 26 °C while the treatment was maintained at 32 °C.

| **Sample ID** | **Number of Reads in the file** | **Number of base pairs** | **Number of reads filtered (% total)** | **Number of base pairs filtered (% total)** |
| --- | --- | --- | --- | --- |
| **T0 control rep1 – R1** | 2,377,136 | 297,142,000 | - | - |
| **T0 control rep1 – R2** | 2,377,136 | 297,142,000 | - | - |
| **T0 control rep2 – R1** | 12,198,374 | 1,524,796,750 | - | - |
| **T0 control rep2 – R2** | 12,198,374 | 1,524,796,750 | - | - |
| **T0 control rep3 – R1** | 5,723,834 | 715,479,250 | - | - |
| **T0 control rep3 – R2** | 5,723,834 | 715,479,250 | - | - |
| **T0 control rep4 – R1** | 11,083,090 | 1,385,386,250 | - | - |
| **T0 control rep4 – R2** | 11,083,090 | 1,385,386,250 | - | - |
| **T0 treatment rep1 – R1** | 2,568,701 | 321,087,625 | - | - |
| **T0 treatment rep1 – R2** | 2,665,675 | 333,209,375 | - | - |
| **T0 treatment rep2 – R1** | 2,665,675 | 333,209,375 | - | - |
| **T0 treatment rep2 – R2** | 3,094,901 | 386,862,625 | - | - |
| **T0 treatment rep3 – R1** | 3,094,901 | 386,862,625 | - | - |
| **T0 treatment rep3 – R2** | 2,587,034 | 323,379,250 | - | - |
| **T0 treatment rep4 – R1** | 2,587,034 | 323,379,250 | - | - |
| **T0 treatment rep4 – R2** | 2,568,701 | 321,087,625 | - | - |
| **TI control rep1 – R1** | 16,791,856 | 1,679,185,600 | 16,791,856 (100.0%) | 1,653,372,636 (98.5%) |
| **TI control rep1 – R2** | 16,791,856 | 1,679,185,600 | 16,791,856 (100.0%) | 1,649,630,971 (98.2%) |
| **TI control rep2 – R1** | 14,197,324 | 1,419,732,400 | 14,197,324 (100.0%) | 1,396,367,687 (98.4%) |
| **TI control rep2 – R2** | 14,197,324 | 1,419,732,400 | 14,197,324 (100.0%) | 1,393,120,125 (98.1%) |
| **TI control rep3 – R1** | 16,120,032 | 1,612,003,200 | 16,120,032 (100.0%) | 1,586,814,250 (98.4%) |
| **TI control rep3 – R2** | 16,120,032 | 1,612,003,200 | 16,120,032 (100.0%) | 1,583,152,244 (98.2%) |
| **TI control rep4 – R1** | 13,723,899 | 1,372,389,900 | 13,723,899 (100.0%) | 1,349,061,963 (98.3%) |
| **TI control rep4 – R2** | 13,723,899 | 1,372,389,900 | 13,723,899 (100.0%) | 1,346,445,168 (98.1%) |
| **TI treatment rep1 – R1** | 14,626,475 | 1,462,647,500 | 14,626,475 (100.0%) | 1,437,401,432 (98.3%) |
| **TI treatment rep1 – R2** | 14,626,475 | 1,462,647,500 | 14,626,475 (100.0%) | 1,430,278,010 (97.8%) |
| **TI treatment rep2 – R1** | 14,800,344 | 1,480,034,400 | 14,800,344 (100.0%) | 1,444,361,473 (97.6%) |
| **TI treatment rep2 – R2** | 14,800,344 | 1,480,034,400 | 14,800,344 (100.0%) | 1,441,981,507 (97.4%) |
| **TI treatment rep3 – R1** | 14,127,073 | 1,412,707,300 | 14,127,073 (100.0%) | 1,386,076,732 (98.1%) |
| **TI treatment rep3 – R2** | 14,127,073 | 1,412,707,300 | 14,127,073 (100.0%) | 1,381,190,756 (97.8%) |
| **TI treatment rep4 – R1** | 13,741,709 | 1,374,170,900 | 13,741,709 (100.0%) | 1,348,892,808 (98.2%) |
| **TI treatment rep4 – R2** | 13,741,709 | 1,374,170,900 | 13,741,709 (100.0%) | 1,345,994,540 (97.9%) |
| **TE control rep1 – R1** | 15,491,084 | 1,549,108,400 | 15,491,084 (100.0%) | 1,527,257,299 (98.6%) |
| **TE control rep1 – R2** | 15,491,084 | 1,549,108,400 | 15,491,084 (100.0%) | 1,522,790,618 (98.3%) |
| **TE control rep2 – R1** | 19,460,394 | 1,946,039,400 | 19,460,394 (100.0%) | 1,918,307,080 (98.6%) |
| **TE control rep2 – R2** | 19,460,394 | 1,946,039,400 | 19,460,394 (100.0%) | 1,910,238,415 (98.2%) |
| **TE control rep3 – R1** | 14,864,560 | 1,486,456,000 | 14,864,560 (100.0%) | 1,463,751,827 (98.5%) |
| **TE control rep3 – R2** | 14,864,560 | 1,486,456,000 | 14,864,560 (100.0%) | 1,458,966,792 (98.2%) |
| **TE control rep4 – R1** | 14,005,929 | 1,400,592,900 | 14,005,929 (100.0%) | 1,383,315,235 (98.8%) |
| **TE control rep4 – R2** | 14,005,929 | 1,400,592,900 | 14,005,929 (100.0%) | 1,376,267,134 (98.3%) |
| **TE treatment rep1 – R1** | 15,060,035 | 1,506,003,500 | 15,060,035 (100.0%) | 1,476,104,491 (98.0%) |
| **TE treatment rep1 – R2** | 15,060,035 | 1,506,003,500 | 15,060,035 (100.0%) | 1,473,530,319 (97.8%) |
| **TE treatment rep2 – R1** | 14,741,991 | 1,474,199,100 | 14,741,991 (100.0%) | 1,434,239,311 (97.3%) |
| **TE treatment rep2 – R2** | 14,741,991 | 1,474,199,100 | 14,741,991 (100.0%) | 1,434,635,658 (97.3%) |
| **TE treatment rep3 – R1** | 14,664,820 | 1,466,482,000 | 14,664,820 (100.0%) | 1,434,157,170 (97.8%) |
| **TE treatment rep3 – R2** | 14,664,820 | 1,466,482,000 | 14,664,820 (100.0%) | 1,432,706,697 (97.7%) |
| **TE treatment rep4 – R1** | 14,579,737 | 1,457,973,700 | 14,579,737 (100.0%) | 1,422,464,994 (97.6%) |
| **TE treatment rep4 – R2** | 14,579,737 | 1,457,973,700 | 14,579,737 (100.0%) | 1,421,284,472 (97.5%) |

**Table S2. Summary Statistics RNASeq data for the Symbiodiniaceae isolate *Cladocopium goreaui* (C1-124)*.*** Sample column demotes the sample ID, which indicates sampling timepoint (start (T0), first time point (TI) or end time point (TE))-control or treatment-replicate-read direction (forward for R1 and reverse for R2). Control was maintained at ca. 26 °C while the treatment was maintained at 32 °C.

| **Sample ID** | **Number of Reads in the file** | **Number of base pairs** | **Number of reads filtered (% total)** | **Number of base pairs filtered (% total)** |
| --- | --- | --- | --- | --- |
| **T0 control rep1 – R1** | 9,462,068 | 1,182,758,500 | - | - |
| **T0 control rep1 – R2** | 9,462,068 | 1,182,758,500 | - | - |
| **T0 control rep2 – R1** | 3,897,857 | 487,232,125 | - | - |
| **T0 control rep2 – R2** | 3,897,857 | 487,232,125 | - | - |
| **T0 control rep3 – R1** | 2,209,286 | 276,160,750 | - | - |
| **T0 control rep3 – R2** | 2,209,286 | 276,160,750 | - | - |
| **T0 control rep4 – R1** | 2,349,864 | 293,733,000 | - | - |
| **T0 control rep4 – R2** | 2,349,864 | 293,733,000 | - | - |
| **T0 treatment rep1 – R1** | 2,569,560 | 321,195,000 | - | - |
| **T0 treatment rep1 – R2** | 2,569,560 | 321,195,000 | - | - |
| **T0 treatment rep2 – R1** | 2,870,295 | 358,786,875 | - | - |
| **T0 treatment rep2 – R2** | 2,870,295 | 358,786,875 | - | - |
| **T0 treatment rep3 – R1** | 2,590,377 | 323,797,125 | - | - |
| **T0 treatment rep3 – R2** | 2,590,377 | 323,797,125 | - | - |
| **T0 treatment rep4 – R1** | 2,656,291 | 332,036,375 | - | - |
| **T0 treatment rep4 – R2** | 2,656,291 | 332,036,375 | - | - |
| **TI control rep1 – R1** | 13,801,356 | 1,380,135,600 | 13,801,356 (100.0%) | 1,355,178,067 (98.2%) |
| **TI control rep1 – R2** | 13,801,356 | 1,380,135,600 | 13,801,356 (100.0%) | 1,352,119,744 (98.0%) |
| **TI control rep2 – R1** | 13,570,294 | 1,357,029,400 | 13,570,294 (100.0%) | 1,337,735,353 (98.6%) |
| **TI control rep2 – R2** | 13,570,294 | 1,357,029,400 | 13,570,294 (100.0%) | 1,334,850,101 (98.4%) |
| **TI control rep3 – R1** | 12,777,581 | 1,277,758,100 | 12,777,581 (100.0%) | 1,256,555,616 (98.3%) |
| **TI control rep3 – R2** | 12,777,581 | 1,277,758,100 | 12,777,581 (100.0%) | 1,253,737,749 (98.1%) |
| **TI control rep4 – R1** | 13,747,568 | 1,374,756,800 | 13,747,568 (100.0%) | 1,350,850,516 (98.3%) |
| **TI control rep4 – R2** | 13,747,568 | 1,374,756,800 | 13,747,568 (100.0%) | 1,348,170,618 (98.1%) |
| **TI treatment rep1 – R1** | 14,166,477 | 1,416,647,700 | 14,166,477 (100.0%) | 1,391,811,621 (98.2%) |
| **Ti treatment rep1 – R2** | 14,166,477 | 1,416,647,700 | 14,166,477 (100.0%) | 1,385,207,945 (97.8%) |
| **TI treatment rep2 – R1** | 14,282,929 | 1,428,292,900 | 14,282,929 (100.0%) | 1,407,546,894 (98.5%) |
| **TI treatment rep2 – R2** | 14,282,929 | 1,428,292,900 | 14,282,929 (100.0%) | 1,402,462,942 (98.2%) |
| **TI treatment rep3 – R1** | 13,153,993 | 1,315,399,300 | 13,153,993 (100.0%) | 1,291,017,961 (98.1%) |
| **TI treatment rep3 – R2** | 13,153,993 | 1,315,399,300 | 13,153,993 (100.0%) | 1,286,415,791 (97.8%) |
| **TI treatment rep4 – R1** | 12,351,282 | 1,235,128,200 | 12,351,282 (100.0%) | 1,215,761,819 (98.4%) |
| **TI treatment rep4 – R2** | 12,351,282 | 1,235,128,200 | 12,351,282 (100.0%) | 1,211,804,111 (98.1%) |
| **TE control rep1 – R1** | 14,084,730 | 1,408,473,000 | 14,084,730 (100.0%) | 1,389,948,935 (98.7%) |
| **TE control rep1 – R2** | 14,084,730 | 1,408,473,000 | 14,084,730 (100.0%) | 1,384,208,018 (98.3%) |
| **TE control rep2 – R1** | 14,113,368 | 1,411,336,800 | 14,113,368 (100.0%) | 1,393,996,684 (98.8%) |
| **TE control rep2 – R2** | 14,113,368 | 1,411,336,800 | 14,113,368 (100.0%) | 1,387,041,798 (98.3%) |
| **TE control rep3 – R1** | 14,621,634 | 1,462,163,400 | 14,621,634 (100.0%) | 1,440,523,358 (98.5%) |
| **TE control rep3 – R2** | 14,621,634 | 1,462,163,400 | 14,621,634 (100.0%) | 1,434,848,111 (98.1%) |
| **TE control rep4 – R1** | 14,517,100 | 1,451,710,000 | 14,517,100 (100.0%) | 1,431,699,730 (98.6%) |
| **TE control rep4 – R2** | 14,517,100 | 1,451,710,000 | 14,517,100 (100.0%) | 1,428,356,984 (98.4%) |
| **TE treatment rep1 – R1** | 13,658,234 | 1,365,823,400 | 13,658,234 (100.0%) | 1,343,570,638 (98.4%) |
| **TE treatment rep1 – R2** | 13,658,234 | 1,365,823,400 | 13,658,234 (100.0%) | 1,335,663,175 (97.8%) |
| **TE treatment rep2 – R1** | 14,061,984 | 1,406,198,400 | 14,061,984 (100.0%) | 1,384,587,223 (98.5%) |
| **TE treatment rep2 – R2** | 14,061,984 | 1,406,198,400 | 14,061,984 (100.0%) | 1,378,072,918 (98.0%) |
| **TE treatment rep3 – R1** | 15,931,877 | 1,593,187,700 | 15,931,877 (100.0%) | 1,569,651,272 (98.5%) |
| **TE treatment rep3 – R2** | 15,931,877 | 1,593,187,700 | 15,931,877 (100.0%) | 1,561,514,444 (98.0%) |
| **TE treatment rep4 – R1** | 13,088,977 | 1,308,897,700 | 13,088,977 (100.0%) | 1,288,285,736 (98.4%) |
| **TE treatment rep4 – R2** | 13,088,977 | 1,308,897,700 | 13,088,977 (100.0%) | 1,285,663,730 (98.2%) |

**Table S3. Summary Statistics RNASeq data for the Symbiodiniaceae isolate *Durusdinium trenchii* (D1a)*.*** Sample column demotes the sample ID, which indicates sampling timepoint (start (T0), first time point (TI) or end time point (TE))-control or treatment-replicate-read direction (forward for R1 and reverse for R2). Control was maintained at ca. 26 °C while the treatment was maintained at 32 °C.

| **Sample ID** | **Number of Reads in the file** | **Number of base pairs** | **Number of reads filtered (% total)** | **Number of base pairs filtered (% total)** |
| --- | --- | --- | --- | --- |
| **T0 control rep1 – R1** | 2,327,730 | 290,966,250 | - | - |
| **T0 control rep1 – R2** | 2,327,730 | 290,966,250 | - | - |
| **T0 control rep2 – R1** | 2,366,193 | 295,774,125 | - | - |
| **T0 control rep2 – R2** | 2,366,193 | 295,774,125 | - | - |
| **T0 control rep3 – R1** | 2,987,563 | 373,445,375 | - | - |
| **T0 control rep3 – R2** | 2,987,563 | 373,445,375 | - | - |
| **T0 control rep4 – R1** | 11,543,797 | 1,442,974,625 | - | - |
| **T0 control rep4 – R2** | 11,543,797 | 1,442,974,625 | - | - |
| **T0 treatment rep1 – R1** | 2,851,502 | 356437750 | - | - |
| **T0 treatment rep1 – R2** | 2,851,502 | 356437750 | - | - |
| **T0 treatment rep2 – R1** | 2,899,542 | 362442750 | - | - |
| **T0 treatment rep2 – R2** | 2,899,542 | 362442750 | - | - |
| **T0 treatment rep3 – R1** | 2,754,308 | 344288500 | - | - |
| **T0 treatment rep3 – R2** | 2,754,308 | 344,288,500 | - | - |
| **T0 treatment rep4 – R1** | 2,637,207 | 329,650,875 | - | - |
| **T0 treatment rep4 – R2** | 2,637,207 | 329,650,875 | - | - |
| **TI control rep1 – R1** | 14,281,445 | 1,428,144,500 | 14,281,445 (100.0%) | 1,403,057,585 (98.2%) |
| **TI control rep1 – R2** | 14,281,445 | 1,428,144,500 | 14,281,445 (100.0%) | 1,399,829,770 (98.0%) |
| **TI control rep2 – R1** | 14,419,544 | 1,441,954,400 | 14,419,544 (100.0%) | 1,419,681,069 (98.5%) |
| **TI control rep2 – R2** | 14,419,544 | 1,441,954,400 | 14,419,544 (100.0%) | 1,416,528,302 (98.2%) |
| **TI control rep3 – R1** | 14,915,702 | 1,491,570,200 | 14,915,702 (100.0%) | 1,467,508,453 (98.4%) |
| **TI control rep3 – R2** | 14,915,702 | 1,491,570,200 | 14,915,702 (100.0%) | 1,461,860,148 (98.0%) |
| **TI control rep4 – R1** | 13,776,252 | 1,377,625,200 | 13,776,252 (100.0%) | 1,355,180,989 (98.4%) |
| **TI control rep4 – R2** | 13,776,252 | 1,377,625,200 | 13,776,252 (100.0%) | 1,351,686,245 (98.1%) |
| **TI treatment rep1 – R1** | 14,438,415 | 1,443,841,500 | 14,438,415 (100.0%) | 1,420,540,435 (98.4%) |
| **Ti treatment rep1 – R2** | 14,438,415 | 1,443,841,500 | 14,438,415 (100.0%) | 1,414,636,367 (98.0%) |
| **TI treatment rep2 – R1** | 15,168,100 | 1,516,810,000 | 15,168,100 (100.0%) | 1,491,534,260 (98.3%) |
| **TI treatment rep2 – R2** | 15,168,100 | 1,516,810,000 | 15,168,100 (100.0%) | 1,487,732,282 (98.1%) |
| **TI treatment rep3 – R1** | 13,560,738 | 1,356,073,800 | 13,560,738 (100.0%) | 1,333,021,999 (98.3%) |
| **TI treatment rep3 – R2** | 13,560,738 | 1,356,073,800 | 13,560,738 (100.0%) | 1,324,845,093 (97.7%) |
| **TI treatment rep4 – R1** | 14,088,773 | 1,408,877,300 | 14,088,773 (100.0%) | 1,385,954,753 (98.4%) |
| **TI treatment rep4 – R2** | 14,088,773 | 1,408,877,300 | 14,088,773 (100.0%) | 1,378,275,470 (97.8%) |
| **TE control rep1 – R1** | 13,386,413 | 1,338,641,300 | 13,386,413 (100.0%) | 1,319,015,979 (98.5%) |
| **TE control rep1 – R2** | 13,386,413 | 1,338,641,300 | 13,386,413 (100.0%) | 1,312,936,031 (98.1%) |
| **TE control rep2 – R1** | 11,827,382 | 1,182,738,200 | 11,827,382 (100.0%) | 1,165,503,722 (98.5%) |
| **TE control rep2 – R2** | 11,827,382 | 1,182,738,200 | 11,827,382 (100.0%) | 1,159,459,417 (98.0%) |
| **TE control rep3 – R1** | 15,917,676 | 1,591,767,600 | 15,917,676 (100.0%) | 1,565,561,297 (98.4%) |
| **TE control rep3 – R2** | 15,917,676 | 1,591,767,600 | 15,917,676 (100.0%) | 1,555,940,390 (97.7%) |
| **TE control rep4 – R1** | 13,792,837 | 1,379,283,700 | 13,792,837 (100.0%) | 1,359,335,635 (98.6%) |
| **TE control rep4 – R2** | 13,792,837 | 1,379,283,700 | 13,792,837 (100.0%) | 1,352,063,607 (98.0%) |
| **TE treatment rep1 – R1** | 17,703,024 | 1,770,302,400 | 17,703,024 (100.0%) | 1,740,449,307 (98.3%) |
| **TE treatment rep1 – R2** | 17,703,024 | 1,770,302,400 | 17,703,024 (100.0%) | 1,733,579,563 (97.9%) |
| **TE treatment rep2 – R1** | 14,155,605 | 1,415,560,500 | 14,155,605 (100.0%) | 1,391,608,785 (98.3%) |
| **TE treatment rep2 – R2** | 14,155,605 | 1,415,560,500 | 14,155,605 (100.0%) | 1,383,676,010 (97.7%) |
| **TE treatment rep3 – R1** | 13,617,652 | 1,361,765,200 | 13,617,652 (100.0%) | 1,339,154,897 (98.3%) |
| **TE treatment rep3 – R2** | 13,617,652 | 1,361,765,200 | 13,617,652 (100.0%) | 1,333,872,700 (98.0%) |
| **TE treatment rep4 – R1** | 14,283,341 | 1,428,334,100 | 14,283,341 (100.0%) | 1,405,001,846 (98.4%) |
| **TE treatment rep4 – R2** | 14,283,341 | 1,428,334,100 | 14,283,341 (100.0%) | 1,397,262,978 (97.8%) |
